# Daily domain-specific time-use composition of physical behaviors and blood pressure

**DOI:** 10.1186/s12966-018-0766-1

**Published:** 2019-01-10

**Authors:** Nidhi Gupta, Mette Korshøj, Dorothea Dumuid, Pieter Coenen, Karen Allesøe, Andreas Holtermann

**Affiliations:** 1 National Research Centre for the Working Environment, Copenhagen, Denmark; 2 Alliance for Research in Exercise, Nutrition, and Activity (ARENA), School of Health Sciences, University of South Australia, Adelaide, South Australia Australia; 3 Department of Public and Occupational Health, Amsterdam Public Health Research Institute, VU University Medical Center, Amsterdam, The Netherlands; 4 Center for Clinical Research and Prevention, Bispebjerg and Frederiksberg Hospital, The Capital Region, Copenhagen, Denmark; 5 Department of Sports Science and Clinical Biomechanics, University of Southern Denmark, Odense, Denmark

**Keywords:** Accelerometry, Domain, Sleep, Physical activity, Sedentary behaviors

## Abstract

**Background:**

Previous research has shown contrasting effects on hypertension for occupational and leisure-time physical behaviors—physical activity and sedentary behavior and time in bed. However, (a) none of these studies have addressed the compositional property of the physical behaviors and (b) most knowledge on the association between domain-specific physical behaviors and hypertension relies upon self-reported physical behaviors information primarily on white-collar worker study samples. We aimed to be the first to disentangle the relationship between technically measured 24-h time-use behaviors in work and leisure domains and blood pressure among blue-collar workers using a compositional data analysis approach.

**Methods:**

Workers (*n* = 669) wore accelerometers to measure daily minutes of work and leisure sedentary time, light physical activity (LPA) and moderate-to-vigorous physical activity (MVPA), and time in bed which were isometrically log-transformed. Cross-sectional linear association between time-use composition and systolic (SBP) and diastolic (DBP) blood pressure were determined using compositional isotemporal substitutions models.

**Results:**

The time-use composition at the work and leisure domains was significantly associated with SBP (F = 4.98, *p* < 0.001) and DBP (F = 2.91, *p* = 0.008). Reallocating sedentary time to remaining behaviors within each domain—work and leisure—was favorably associated with SBP. Similar results were observed when reallocating time in bed from the remaining leisure behaviors. Results for reallocating time to/from MVPA and LPA at both domains were non-significant. Results regarding all physical behaviors for DBP were generally non-significant.

**Conclusions:**

Time-use composition of physical behaviors at work and leisure is associated with blood pressure among blue-collar workers. At both domains, reallocating sedentary time to remaining behaviors, especially to time in bed at leisure may reduce blood pressure. Our results, based on a compositional data approach, can be used to better design accurate and comprehensive time-use recommendations both at work and leisure for high-risk groups like blue-collar workers.

**Electronic supplementary material:**

The online version of this article (10.1186/s12966-018-0766-1) contains supplementary material, which is available to authorized users.

## Background

Hypertension is an established risk factor for cardiovascular diseases such as stroke [1] and myocardial infarction [1]. In 2015, 21% of the global population was diagnosed with hypertension [2] and alarmingly, it may increase up to 60% by 2025 [3]. Therefore, knowledge of effective preventive strategies for hypertension is needed, especially among high-risk populations, such as blue-collar workers [4, 5].

Physical behaviors such as standing, walking, and sedentary behavior are associated with blood pressure [6–8]. Research has shown elevated blood pressure among those who are sedentary [9] and/or physically inactive [10], which reduced among those breaking up their sedentary behavior [11] or being physically active [12]. Moreover, sleep duration that is too short or long has also been shown to increase the risk of hypertension [13].

Recent evidence suggests a contrasting effect of work and leisure physical behaviors on blood pressure [5, 7, 14–17]. This might be explained by differences in intensity, duration, and frequency of activities at work and leisure [18]. In general, work physical activity is performed at lower intensities and for longer durations [18] than leisure physical activity. Thus, work physical activity may induce a higher total cardiovascular load without reaching sufficient intensity of physical activity to increase cardiorespiratory fitness, and with limited time and decision latitude to achieve sufficient recovery [4, 18] compared to leisure physical activity. Hence, it might be necessary to differentiate between work and leisure behaviors when investigating their association with blood pressure.

Time spent in physical behaviors during work, leisure and sleep constitute mutually exclusive components of the complete day (i.e., 24 h). As such, it is impossible to increase time in one behavior without decreasing time in at least one other behavior within that day. Health impacts associated with an increase in one behavior may depend on which behavior it is replacing. The *compositional* nature of these data imposes important implications for statistical analysis and interpretation which has been addressed by very few studies only [6, 8, 19, 20], investigating the association with blood pressure. For example, one study using compositional data analysis found that reallocating sedentary time with time in bed and MVPA is beneficially associated with hypertension [8]. However, none of these studies have investigated if these associations differ when separating between work and leisure using a compositional data analysis (CoDA) approach. Therefore, there is a need to apply CoDA methodology to investigate the domain-specific association between physical activity and blood pressure.

Most previous studies on preventing hypertension have (a) used self-reported information of physical behaviors at work and leisure [17, 21, 22] which has shown to be inaccurate and biased [23, 24] (b) and/or are centered around the general population or white-collar workers with little focus on lower socioeconomic position groups, like blue-collar workers. These blue-collar workers are generally exposed to high physical work demands (e.g. heavy lifting and static standing) which can increase blood pressure [5], while spending most leisure time (i.e, non-work time) being sedentary [25]. Thus, the 24-h physical behaviors profile for blue-collar workers can be very different from white-collar workers, mostly being sedentary at work and more physically active at leisure.

In this study, we aimed to investigate the association between technically measured physical behaviors during the work and leisure domain and blood pressure among blue-collar workers using a compositional data analysis approach.

## Methods

This study is based on cross-sectional data from the Danish PHysical ACTivity cohort with Objective measurements (DPHACTO) that aims to determine the prospective association between technically measured physical behaviors at work and leisure and monthly measurements of musculoskeletal pain primarily among blue-collar workers (see more details here; 26). Workers from 15 Danish manufacturing, cleaning, and transport workplaces were recruited between 2011 and 2013. Workers were excluded if pregnant, had fever, or were allergic to adhesives. Details about the study protocol, recruitment, and inclusion and exclusion criteria can be found elsewhere [26]. Conducting and reporting of this study has been done in accordance to STROBE statement guidance (Additional file 2).

Of the 2107 eligible workers invited to participate, 1119 consented to participate. Workers were asked to fill in a web-questionnaire and participate in health-related tests and accelerometry. Cross-sectional data in DPHACTO were collected between 2012 and 2013.

### Measurement of daily work and leisure physical behaviors

Workers were instructed to attach a tri-axial Actigraph accelerometer (GT3X+, USA) on their right thigh for 24-h for four consecutive days. Simultaneously, workers were asked to fill-in a short diary noting the time they started and ended work, went and got out of bed, and time of reference measurement (i.e., standing in the upright position for 15 s). Further details of measurements of physical behaviors have been provided elsewhere [27].

An Acti4 program was used to identify periods spent in various physical behaviors (i.e., sedentary behavior, walking slow and walking fast, running, cycling, and stair-climbing) [28]. A description of this program technically detecting time spent in each physical behavior is provided elsewhere [27, 28]. Time spent standing and slow walking was merged to calculate LPA, while fast walking, running, cycling and stair-climbing time was merged to calculate MVPA. Time in bed was defined as the period between going to and getting out of bed. Work period was defined as hours spent in primary occupation. Leisure period was defined as time within a day except for work time and time in bed.

All non-working days and accelerometer non-wear periods were excluded according to previously defined criteria [27]. Workers who had measurements of valid work, leisure and time in bed on at least one day were included in further analyses. Criteria of a valid work and leisure period and valid time in bed period are explained elsewhere [29]**.**

For the analyses, the mean time spent sedentary, in LPA, and MVPA measured on all valid days and a median of all valid time in bed periods was calculated for each worker. We chose these statistics based on the distribution of the data.

### Systolic and diastolic blood pressure

Resting blood pressure measurements were performed during working hours and on the first day of accelerometry. Workers were asked to rest in a seated position for five minutes with their back supported, legs uncrossed and arm supported. Blood pressure was measured on the left arm three times at regular 1–2-min intervals with the Omron M6 Comfort. The average of the last two recordings was used.

### Measurement of confounders

Potential confounders were chosen a priori based on the literature on the association between physical activity and sedentary behavior and blood pressure [30, 31]. Age and sex of the workers were determined using their unique civil registration number. Age was operationalized in two groups; ≤45 years and > 45 years. Body mass index (BMI) was determined by dividing objectively measured weight (kg) by height squared (m^2^), collapsing it into two categories: < 30 kg/m^2^ and ≥ 30 kg/m^2^ [8]. Time spent lifting/carrying at work was determined using a single item with 6 responses ranging from ‘almost all the time’ to ‘never’ [27]. Workers were also asked if they take prescribed medication intake due to hypertension, depression and heart disease**.** Poor dietary intake was determined using a single item “How often do you usually eat Fastfood, pizza, burger, shawarma,” with four responses from ‘Every day’ to ‘Rare’ [8]. Smoking status and alcohol intake information was collected using single items [8]. Information about the job sector of the workers was obtained from the payroll. Two items were used to determine influence at work [32] and another two to determine social support at work [32]. The responses for both variables were converted to a 0 to 100% scale where 0% indicates low influence at work or low social support at work [8, 32].

### Statistical analysis

The statistical analyses were performed in R software (version 3.3.2; 2016-10-31) using the package ‘Compositions’ [33]. Because time-use data are compositional in nature, compositional data analysis (CoDA) was used [6, 34–36]. The first step was to express the time-use composition as a set of isometric log ratios (*ilrs*). The time-use composition consisted of seven behaviors (work and leisure sedentary behavior, work and leisure LPA, work and leisure MVPA, and time in bed), resulting in six *ilr*s.

### Main analysis

Multiple linear regression was used to determine the association between systolic blood pressure (SBP) or diastolic blood pressure (DBP) and the time-use composition (expressed as *ilr*s). First, these analyses were adjusted only for age and gender — crude model. Later, these analyses were further adjusted for BMI, smoking, alcohol intake, poor dietary intake, time spent lifting/carrying at work, and prescribed medicine intake — adjusted model. To interpret the *beta* coefficients, compositional isotemporal substitution analyses were used [8]. The multiple linear regression models (adjusted models) were used to predict the difference in blood pressure for varying time reallocations among parts of the time-use composition. For this analysis, the sample mean composition was used as the reference or baseline composition. From there, the new compositions were generated by increasing/decreasing duration in one behavior and simultaneously taking out/distributing that duration equally from/to the remaining behaviors, to maintain a daily total of 1440 min following the procedure detailed elsewhere [8] also known as *one-to-remaining reallocation*. Because the number of working hours is generally fixed, we performed substitution models within each domain (work or leisure) instead of performing inter-domain reallocations. For example, sedentary time at work was incrementally decreased by 30 min while these 30 min were distributed equally to remaining behaviors at work while keeping the leisure behaviors constant. The differences in predicted blood pressure associated with the reallocation of time between behaviors were plotted to visually display the relationship between the predicted differences in blood pressure associated with a difference in each behavior relative to other behaviors within each domain. The 95% confidence intervals of the predicted differences in blood pressure corresponding to each reallocation were also calculated and are presented in Appendix A and Appendix B for SBP and DBP, respectively.

### Sensitivity analysis

In the main analysis, we did not adjust for psychosocial work factors due to too many (*n* = 208) missing values in these variables. The missing values were due to a technical error that occurred while participants were answering the web-questionnaire. As a result, workers from three participating companies could not report information on these factors. Therefore, we performed a complete case analysis on a subsample (*n* = 461) where information on psychosocial work factors was also available. In this subsample analysis, we compared the results of the model with and without adjusting for the following psychosocial work factors – influence and social support at work.

## Results

**Fig. 1:**
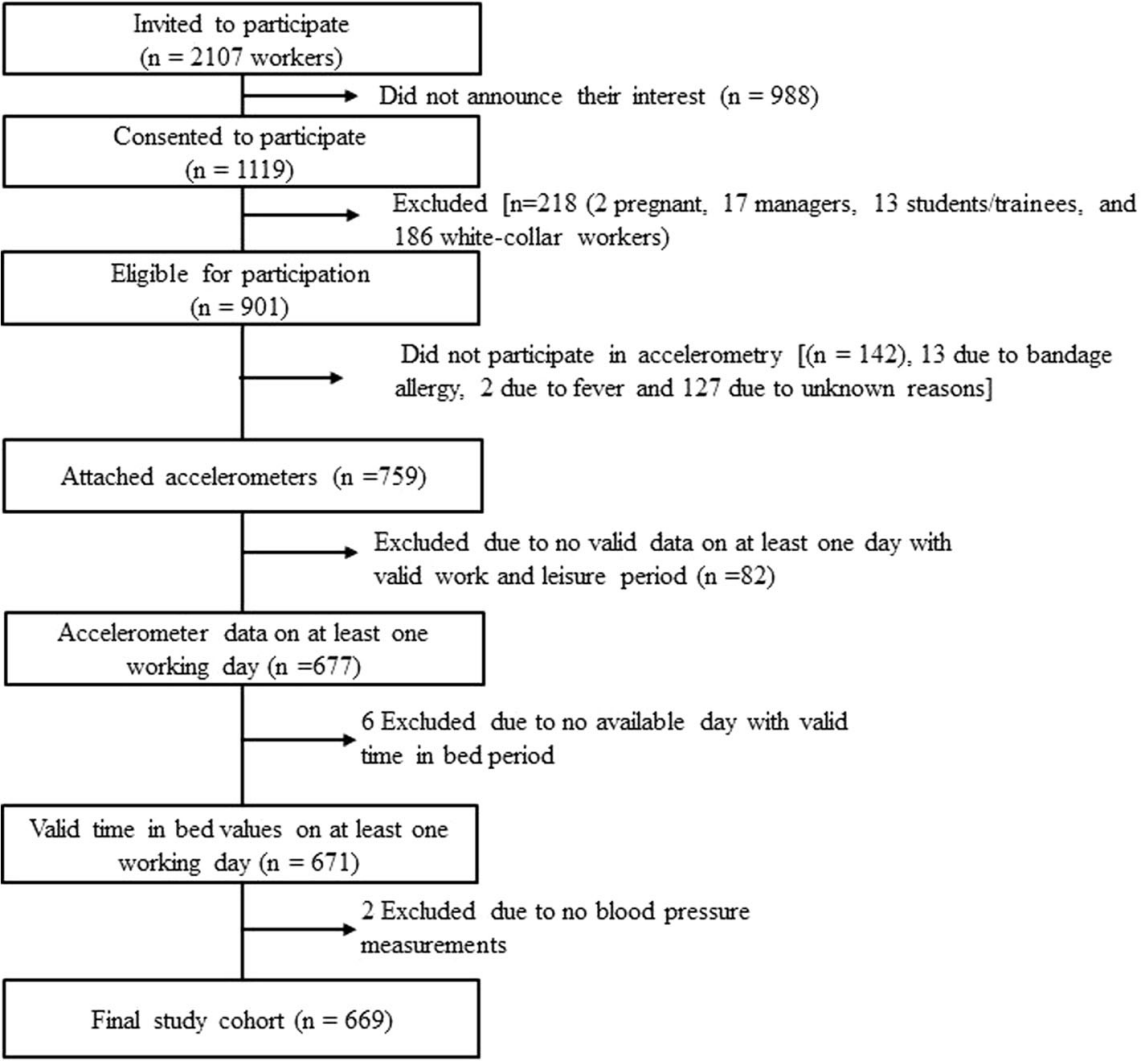
Flow of the participants in this study

Out of 1119 workers consenting to participate, this study included those who were measured for at least one workday including valid accelerometer-based measurements at work, during leisure and in bed and had measurements of blood pressure yielding a final analytical sample of 669 men and women (Fig. 1). On average, workers had 2.7 (SD = 0.9) valid measured days with average wear period of 23.4 ± 1.3 h per day.

Descriptive statistics of the blue-collar workers involved in the analysis are shown in Table 1.

**Table 1:** Description of the 669 blue-collar workers included in the analyses

Statistic	N	%	Mean	St. Dev.
Age (years)	669		45.1	9.9
≤45 yrs	306	46		
> 45 yrs	363	54		
Males	365	55		
Job groups				
Cleaners	130	19		
Manufacturing workers	479	72		
Transport workers	60	9		
BMI (kg/m^2^)	656		27.5	4.9
< 30	481	73		
≥ 30	175	27		
Occupational carry/lift duration (1–6)	667		3.5	1.4
Medication intake				
Yes	108	16		
No	558	84		
Influence at work (0–100%)	461		61.6	26.5
Social support at work (0–100%)	461		78.1	16.5
Poor dietary habit (1–4)	655		2.4	1.2
Alcohol intake	626		4.4	6.3
Smokers	197	30		
Systolic BP (mmHg)	669		133.7	14.9
Diastolic BP (mmHg)	669		83.7	10.4
Accelerometry-based physical behaviors; compositional means (mins/day)^a^				
Work				
Sedentary	669		126	
LPA	669		240	
MVPA	669		67	
Leisure				
Sedentary	669		351	
LPA	669		156	
MVPA	669		44	
Time in bed	669		456	

^a^the multivariate spread of compositional data is described in a variation matrix (Additional file 1)

Additional file 1 shows the pairwise variation between physical behaviors. The variability of the ratios of the physical behaviors at work was generally higher than at leisure. Especially, at both domains, the highest variance was observed for the ratio of LPA and MVPA with sedentary time.

### Results of the main analysis

The 24-h composition of time spent in physical behaviors at work and leisure was a significant predictor of SBP (crude model: F = 4.75, *p* < 0.001; adjusted model: F = 4.98, *p* < 0.001) and DBP (crude model: F = 2.74, *p* = 0.01; adjusted model: F = 2.91, *p* = 0.008).

**Fig. 2:**
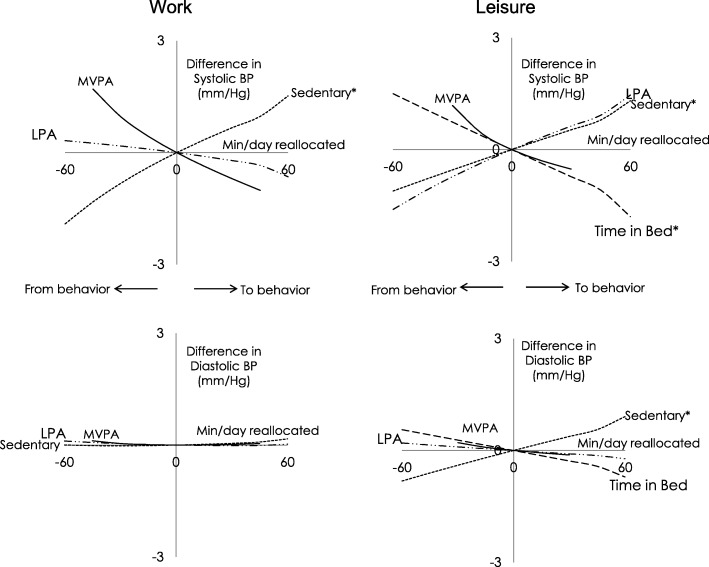
Estimated difference in systolic and diastolic blood pressure associated with one-to-remaining reallocations of physical behaviors during work or leisure among 671 blue-collar workers; X-axis represents the number of minutes reallocated in a behavior to remaining behavior within each domain; * significant at *p* < 0.05; BP = blood pressure. The confidence intervals of the estimates are presented in the Appendix A and Appendix B

Figure 2 shows the results of the compositional isotemporal reallocations based on adjusted models estimates. At both work and during leisure, reallocating time from sedentary behaviors to the remaining behaviors in that domain, was significantly and favorably (i.e., showing a reduction) associated with SBP. For example, reallocating 60 min from work sedentary time to the remaining work behaviors was associated with lower SBP of − 1.9 (95% CI = − 3.0: -0.8) mmHg, and during leisure, the same reallocation was also associated with lower SBP of − 1.1 (− 2.1: − 0.2) mmHg.

For DBP, the associations were only statistically significant for reallocating leisure sedentary time to remaining leisure behaviors, and they were favorable. However, estimated differences in DBP for reallocations of the time were modest. For example, reallocating 60 min from leisure sedentary time to other leisure behaviors was associated with lower DBP of − 0.8 (− 1.5: − 0.1).

Reallocating time from LPA to other behaviors during leisure was favorably but non-statistically significant associated with SBP. For example, reallocating 60 min of leisure LPA to remaining behaviors during this domain was associated with lower SBP of − 1.6 mmHg. This reallocation during work time was weakly unfavorable and non-statistically significant. None of these associations were statistically significant for DBP.

Reallocating more time to bed from other behaviors during leisure was favorably associated with SBP. Reallocating 60 min more to time in bed from other leisure behaviors was associated with lower SBP of − 1.4 (− 2.5: − 0.4). No statistically significant associations were observed for DBP.

Results of reallocating time between MVPA and other behaviors in both domains were statistically non-significant for both SBP and DBP.

Regarding the assumptions of the multivariate linear regression analyses, the residuals were normally distributed, there was no pattern of non-linearity in the residuals and heteroscedasticity were minimal.

### Results of the sensitivity analysis

In the complete case analysis of 461 workers, we found almost identical results when we additionally adjusted for the psychosocial work factors of social support and influence at work (without additional adjustment: SBP: F = 2.95, *p* = 0.008, DBP: F = 1.91, *p* = 0.08; with additional adjustment: SBP: F = 2.96, *p* = 0.008, DBP: F = 1.91, *p* = 0.08).

## Discussion

We were the first to utilize CoDA methodology to investigate the domain-specific association between time-use composition of daily physical behaviors and blood pressure in blue collar workers. The composition of daily physical behaviors was significantly associated with SBP and DBP. At work, SBP was significantly and favorably associated (i.e. lower) with reallocating sedentary time to remaining behaviors. At leisure, similar favorable associations were observed for reallocating sedentary time to remaining behaviors, especially to time in bed. Associations with DBP were generally weak.

Reallocating sedentary time to remaining behaviors within work and leisure domains was favorably associated with SBP. We also observed similar results for DBP but only statistically significant for leisure time sedentary behavior. Overall, the favorable association of blood pressure with reducing sedentary time by increasing time in the other behaviors was similar, irrespective of domain. Specifically, we found that replacing 60 min of sedentary time at work with remaining physical behaviors was associated with almost **≈**2 mmHg SBP which can be interpreted to be a clinically relevant difference [37]. At leisure, this effect appeared to be smaller (− 1.1 mmHg). Numerous studies have found leisure sedentary time to be unfavorable for hypertension [6], cardiorespiratory outcomes [38] and all-cause mortality [39]. A few studies have investigated the differential associations of work and leisure sedentary time and blood pressure [14–16]. Our results are in line with some of these studies [15, 16]. A very few studies using CoDA [6, 8, 19, 20] reported unfavorable or null associations between sedentary time (relative to other behaviors) and blood pressure. However, these studies did mostly not consider time spent in bed or asleep [6, 19], and none of them investigated domain-specific associations of physical behaviors and blood pressure [6, 8, 19, 20].

We observed that the results of reallocating time between MVPA and other behaviors for both domains and blood pressure were statistically non-significant. This is in contrast to previous research indicating beneficial health outcomes associated with leisure MVPA and non-beneficial health outcomes associated with work MVPA among blue-collar workers [40, 41]. A plausible reason behind non-beneficial health outcomes for MVPA at work is that it involves lifting and carrying heavy weights and awkward postures, with little possibility of breaks between activities [18]. As blue-collar workers also typically have low cardiorespiratory fitness [42], occupational MVPA may be strenuous [5, 41]. We did not observe these associations clearly, and therefore we recommend similar future studies to confirm these results.

Reallocating time to bed from other leisure behaviors was associated with lower blood pressure. Specifically, increasing time in bed by decreasing time in remaining physical behaviors at leisure was associated with a 1.4 mmHg decrease in SBP. This concurs with studies reporting a beneficial association between sleep duration and cardiovascular health among the general [43, 44] and working population [8]. Previous research among the general population has also reported a U-shaped association between sleep time and blood pressure [13]. Specifically, individuals sleeping for either ≤5 h or > 9 h/day are generally at risk of hypertension [13]. We did not observe this in our dataset, probably because blue-collar workers are generally engaged in physically strenuous jobs, possibly requiring longer recovery times during leisure compared to the general population. Similar future studies are required to confirm the linearity of associations between sleep duration (relative to other physical behaviors) and blood pressure among blue-collar workers.

Reallocating time between LPA and other behaviors during both domains were non-significantly associated with SBP and DBP. However, the corresponding estimates for leisure LPA seemed clinically relevant [37] — indicating that reallocating LPA time to other leisure behaviors tended to be associated with lower SBP. Conversely, corresponding estimates for work LPA seemed weak. The results of leisure time LPA may seem surprising as earlier work has shown that replacing the sedentary behavior with light to moderate intensity walking lowers blood pressure [11]. However, the previous overall evidence on the effects of LPA at work and leisure on blood pressure is not conclusive [41]. Due to non-significant findings of our study, we recommend conducting similar future studies to confirm our results and underline the importance of considering all domains, intensities, and postures of physical activities in a compositional manner.

The key implication of our findings points towards possible prevention of hypertension by modifying the daily time-use composition at both the work and leisure domain. The choice of reallocation strategy can be dependent on the contextual domain. For example, if it is feasible to conduct a workplace intervention, an optimum strategy may be to reallocate 60 min of sedentary time, a feasible and realistic change according to a previous meta-analysis on workplace interventions [45], by remaining behaviors at work (e.g. walking, non-stationary standing) and especially to MVPA at work (e.g. brisk walking, walking on stairs). At leisure, a feasible strategy may be to decrease 60 min of sedentary time by increasing time in bed or MVPA. However, when adopting these reallocation strategies, it is important to consider possible constraints during the various time-use domains. For example, it may be difficult for a cleaner to replace standing tasks with sedentary tasks at work, while it may be difficult for an individual to avoid standing (for example, cooking time) during leisure due to family commitments.

### Strengths and limitations

This study is the first to use CoDA methodology to understand domain-specific time-use composition of physical behaviors. Thus our study presents an analytical base for future studies to address domain-specific associations of compositional physical behaviors with health outcomes. Another strength of our study is the device-based measurement of physical behaviors which is less erroneous and less susceptible to measurement errors than self-reports. As the devices used in this study were waterproof, workers could wear them while showering or swimming unlike in previous research [46]. Consequently, workers wore the devices for 23.4 ± 1.3 h/day. Acti4 software derives time spent in physical behaviors based on posture detections rather than on count per minute based thresholds, which have been heavily criticized for being unable to distinguish between some postures [for example sitting and standing postures [47]].

One limitation of the study is the cross-sectional design which does not allow us to make inferences about causality between time use and blood pressure. We used a conventional method to measure resting blood pressure that is a common practice in many studies and has shown to predict cardiovascular risk for mortality [48, 49]. However, resting blood pressure can be affected due to time of day, hydration level, recent mental stressors, smoking, and bouts of strenuous physical activity [50]. Therefore, ambulatory blood pressure measurements are preferable to resting blood pressure measurements [50, 51] and hence, we recommend future studies to confirm our results using ambulatory blood pressure measurements. Another limitation is the use of time in bed as a proxy for sleep. Future studies should divide time in bed into wakeful and non-wakeful periods. Our study was conducted only on blue-collar workers. Thus, the generalization of the results from this study should be made with care, especially for white-collar workers. We performed 4-day accelerometry that may, in some cases, not capture the usual physical activity of the participants. However, a number of studies have suggested that 4-day accelerometry is sufficient to reliably estimate habitual time spent in various physical behaviors [52, 53]. Additionally, we cannot ignore the fact that there may be residual confounding due to unmeasured factors.

## Conclusion

Time-use composition of various physical behaviors at work and leisure and time in bed was associated with SBP and DBP. At work, favorable associations were observed when sedentary time was reallocated to remaining behaviors. During leisure, it seems beneficial to reallocate time from sedentary behavior to remaining behaviors, especially to time in bed. Behavioral modification strategies based on reallocations of time within the composition of physical behaviors at work and leisure provide useful knowledge for the prevention of hypertension, especially among this high-risk group of lower socioeconomic blue-collar workers.

### Additional file

Additional file 1: Variation matrix showing the variation of each physical behavior relative to remaining behaviors at work and leisure domains among 671 blue-collar workers. (DOCX 13 kb)

Additional file 2: STROBE Statement—checklist of items that should be included in reports of observational studies. (DOCX 22 kb)

## Appendix A

**Table 2:** Estimated difference in systolic blood pressure (95% confidence intervals) associated with one-to-remaining reallocations of physical behaviors at work or leisure among 671 blue-collar workers

Difference in behavior (min/day)	Estimated difference in SBP (mmHg) (95% CI)							
	Sedentary to remaining^a^		LPA to remaining^a^		MVPA to remaining^a^		Time in bed to remaining^a^	
Work								
− 90	**−3.6**	**(− 5.8:-1.4)**	0.4	(−1.6:2.4)				
−75	**−2.6**	**(− 4.2:-1.1)**	0.4	(− 1.3:2.0)				
−60	**−1.9**	**(−3.0:-0.8)**	0.3	(−1.0:1.6)				
−45	**−1.3**	**(−2.1:-0.5)**	0.3	(−0.7:1.2)	1.7	(−1.4:4.8)		
−30	**− 0.8**	**(−1.3:-0.3)**	0.2	(− 0.5:0.8)	1.0	(− 0.7:2.6)		
−15	**−0.4**	**(− 0.6:-0.2)**	0.1	(− 0.2:0.4)	0.4	(− 0.3:1.2)		
0	**0.0**	**(0.0:0.0)**	0.0	(0.0:0.0)	0.0	(0.0:0.0)		
15	**0.4**	**(0.1:0.6)**	−0.1	(−0.4:0.2)	−0.4	(−1.0:0.2)		
30	**0.7**	**(0.3:1.1)**	−0.2	(−0.9:0.5)	− 0.7	(−1.8:0.4)		
45	**1.0**	**(0.4:1.6)**	−0.3	(−1.4:0.7)	− 1.0	(−2.6:0.6)		
60	**1.3**	**(0.5:2.0)**	−0.5	(−1.9:0.9)				
75	**1.5**	**(0.5:2.5)**	−0.6	(−2.5: 1.2)				
90	**1.8**	**(0.6:2.9)**	− 0.8	(−3.1:1.5)				
Leisure								
−90	**−1.7**	**(−3.2:-0.2)**	− 2.8	(−5.8:0.3)			**2.3**	**(0.7:3.9)**
−75	**−1.4**	**(−2.6:-0.2)**	−2.1	(−4.5:0.2)			**1.9**	**(0.6:3.2)**
−60	**−1.1**	**(−2.1:-0.2)**	− 1.6	(−3.4:0.2)			**1.5**	**(0.4:2.6)**
−45	**−0.8**	**(−1.5:-0.1)**	−1.1	(−2.4:0.1)			**1.1**	**(0.3:1.9)**
−30	**−0.5**	**(−1.0:-0.1)**	− 0.7	(− 1.5:0.1)	1.2	(−2.0:4.4)	**0.7**	**(0.2:1.3)**
−15	**−0.3**	**(−0.5:0.0)**	− 0.3	(− 0.7:0.0)	0.4	(− 0.7:1.6)	**0.4**	**(0.1:0.6)**
0	**0.0**	**(0.0:0.0)**	0.0	(0.0:0.0)	0.0	(0.0:0.0)	**0.0**	**(0.0:0.0)**
15	**0.3**	**(0.0:0.5)**	0.3	(0.0:0.7)	−0.3	(−1.1:0.5)	**−0.4**	**(− 0.6:-0.1)**
30	**0.5**	**(0.1:1.0)**	0.6	(0.0:1.3)	−0.5	(−2.0:1.0)	**−0.7**	**(−1.2:-0.2)**
45	**0.8**	**(0.1:1.5)**	0.9	(−0.1:1.9)			**−1.1**	**(−1.8:-0.3)**
60	**1.1**	**(0.2:2.0)**	1.2	(−0.1:2.5)			**−1.4**	**(−2.5:-0.4)**
75	**1.3**	**(0.2:2.4)**	1.5	(−0.1:3.0)			**−1.8**	**(−3.1:-0.5)**
90	**1.6**	**(0.2:2.9)**	1.7	(−0.1:3.5)			**−2.2**	**(−3.7:-0.6)**

Results in bold are considered statistically significant because the confidence interval did not include 0. ^a^remaining means those behaviors besides one which is getting replaced within each domain. For example sedentary to remaining at work means reallocation between sedentary time and time spent on LPA and MVPA at work. Similarly, MVPA to remaining at leisure means reallocations between MVPA time and time spent on LPA, sedentary and in bed. LPA = light physical activity, MVPA = moderate-to-vigorous physical activity

## Appendix B

**Table 3:** Estimated difference in diastolic blood pressure (95% confidence intervals) associated with one-to-remaining reallocations of physical behaviors at work or leisure among 671 blue-collar workers

Difference in behavior (min/day)	Estimated difference in DBP (mmHg) (95% CI)							
	Sedentary to remaining^a^		LPA to remaining^a^		MVPA to remaining^a^		Time in bed to remaining^a^	
Work								
−90	0.1	(−1.5:1.7)	0.2	(− 1.2:1.7)				
−75	0	(−1.1:1.2)	0.2	(− 1:1.3)				
−60	0	(−0.8:0.8)	0.1	(−0.8:1)				
−45	0	(−0.6:0.5)	0.1	(−0.6:0.8)	0.1	(−2.1:2.3)		
−30	0	(−0.4:0.3)	0	(−0.4:0.5)	0	(−1.2:1.2)		
− 15	0	(− 0.2:0.1)	0	(−0.2:0.3)	0	(−0.5:0.5)		
0	0	(0:0)	0	(0:0)	0	(0:0)		
15	0	(−0.1:0.2)	0	(− 0.2:0.2)	0	(− 0.4:0.4)		
30	0	(−0.2:0.3)	0	(−0.5:0.5)	0	(−0.8:0.8)		
45	0.1	(−0.3:0.5)	0	(−0.8:0.7)	0	(−1.1:1.2)		
60	0.2	(−0.5:0.9)	0	(−1.3:1.3)				
75	0.1	(−0.4:0.7)	0	(−1:1)				
90	0.2	(−0.6:1.1)	0.1	(−1.6:1.7)				
Leisure								
−90	**−1.3**	**(−2.3:-0.2)**	0.3	(−1.9:2.5)			0.8	(−0.3:2)
−75	**−1**	**(− 1.9:-0.2)**	0.3	(− 1.4:1.9)			0.7	(−0.3:1.6)
−60	**−0.8**	**(−1.5:-0.1)**	0.2	(−1.1:1.5)			0.6	(−0.2:1.3)
−45	**−0.6**	**(−1.1:-0.1)**	0.1	(−0.7:1)			0.4	(−0.1:1)
−30	**−0.4**	**(− 0.7:-0.1)**	0.1	(−0.5:0.7)	0.2	(−2.1:2.5)	0.3	(−0.1:0.7)
−15	**−0.2**	**(−0.4:0)**	0	(−0.2:0.3)	0.1	(−0.8:0.9)	0.1	(0:0.3)
0	**0**	**(0:0)**	0	(0:0)	0	(0:0)	0	(0:0)
15	**0.2**	**(0:0.4)**	0	(−0.3:0.2)	−0.1	(− 0.7:0.5)	−0.1	(− 0.3:0)
30	**0.4**	**(0.1:0.7)**	−0.1	(−0.6:0.4)	− 0.1	(−1.2:0.9)	−0.3	(− 0.6:0.1)
45	**0.6**	**(0.1:1)**	−0.1	(−0.8:0.6)			−0.4	(−1:0.1)
60	**0.7**	**(0.1:1.4)**	−0.2	(−1.1:0.7)			−0.6	(−1.3:0.2)
75	**0.9**	**(0.1:1.7)**	−0.2	(−1.3:0.9)			−0.7	(−1.6:0.2)
90	**1.1**	**(0.1:2)**	−0.3	(−1.6:1)			−0.9	(−1.9:0.2)

Results in bold are considered statistically significant because the confidence interval did not include 0. ^a^remaining means those behaviors besides one which is getting replaced within each domain. For example sedentary to remaining at work means reallocation between sedentary time and time spent on LPA and MVPA at work. Similarly, MVPA to remaining at leisure means reallocations between MVPA time and time spent on LPA, sedentary and in bed. LPA = light physical activity, MVPA = moderate-to-vigorous physical activity.

## Abbreviations

BMI: Body mass index
CoDA: Compositional data analysis
DBP: Diastolic blood pressure
LPA: Light physical activity
MVPA: Moderate-to-vigorous physical activity
SBP: Systolic blood pressure
