# Supplementary material for: Daily domain-specific time-use composition of physical behaviors and blood pressure
Source: Int J Behav Nutr Phys Act. 2019 Jan 10;16:4. doi: 10.1186/s12966-018-0766-1 (PMC6327498; doi:10.1186/s12966-018-0766-1)
Supplement: Supplementary file 1 — Variation matrix showing the variation of each physical behavior relative to remaining behaviors at work and leisure domains among 671 blue-collar workers. (DOCX 13 kb) [file 12966_2018_766_MOESM1_ESM.docx]

Additional file

Variation matrix showing the variation of each physical behavior relative to remaining behaviors at work and leisure domains among 671 blue-collar workers

| Variable |  | Work |  |  | Leisure |  |  |
| --- | --- | --- | --- | --- | --- | --- | --- |
| Work | Sedentary | LPA | MVPA | Sedentary | LPA | MVPA | Time in bed |
| Sedentary | 0.00 | 1.37 | 1.02 | 0.72 | 0.94 | 1.05 | 0.66 |
| LPA | 1.37 | 0.00 | 0.26 | 0.23 | 0.35 | 0.44 | 0.18 |
| MVPA | 1.02 | 0.26 | 0.00 | 0.27 | 0.36 | 0.35 | 0.22 |
| Leisure |  |  |  |  |  |  |  |
| Sedentary | 0.72 | 0.23 | 0.27 | 0.00 | 0.29 | 0.43 | 0.10 |
| LPA | 0.94 | 0.35 | 0.36 | 0.29 | 0.00 | 0.18 | 0.19 |
| MVPA | 1.05 | 0.44 | 0.35 | 0.43 | 0.18 | 0.00 | 0.28 |
| Time in bed | 0.66 | 0.18 | 0.22 | 0.10 | 0.19 | 0.28 | 0.00 |

Each cell of the table shows the variance of pairwise log-ratios between behaviors stated by the row (numerator) and the column (denominator). Variance close to 0 indicates higher proportionality between two behaviors. For example, during leisure, the variance of ln (LPA/MVPA) is 0.18, suggesting that these two behaviors are highly proportional, or co-varying. LPA = light physical activity, MVPA = moderate-to-vigorous physical activity
