# Supplementary material for: Daily domain-specific time-use composition of physical behaviors and blood pressure
Source: Int J Behav Nutr Phys Act. 2019 Jan 10;16:4. doi: 10.1186/s12966-018-0766-1 (PMC6327498; doi:10.1186/s12966-018-0766-1)
Supplement: Supplementary file 2 — STROBE Statement—checklist of items that should be included in reports of observational studies. (DOCX 22 kb) [file 12966_2018_766_MOESM2_ESM.docx]

STROBE Statement—checklist of items that should be included in reports of observational studies

|  | Item No | Recommendation |
| --- | --- | --- |
| **Title and abstract** | 1 | (*a*) Indicate the study’s design with a commonly used term in the title or the abstract (✓ **indicated in the abstract**) |
|  |  | (*b*) Provide in the abstract an informative and balanced summary of what was done and what was found **(✓ page 3)** |
| Introduction | | |
| Background/rationale | 2 | Explain the scientific background and rationale for the investigation being reported **(✓ page 6–7)** |
| Objectives | 3 | State specific objectives, including any prespecified hypotheses **(✓page 7)** |
| Methods | | |
| Study design | 4 | Present key elements of study design early in the paper **(✓page 8)** |
| Setting | 5 | Describe the setting, locations, and relevant dates, including periods of recruitment, exposure, follow-up, and data collection **(✓page 8)** |
| Participants | 6 | *Cross-sectional study*—Give the eligibility criteria, and the sources and methods of selection of participants **(✓page 8)** |
|  |  |  |
| Variables | 7 | Clearly define all outcomes, exposures, predictors, potential confounders, and effect modifiers. Give diagnostic criteria, if applicable **(✓page 8–10)** |
| Data sources/ measurement | 8* | For each variable of interest, give sources of data and details of methods of assessment (measurement). Describe comparability of assessment methods if there is more than one group **(✓page 8–10)** |
| Bias | 9 | Describe any efforts to address potential sources of bias **(N/A)** |
| Study size | 10 | Explain how the study size was arrived at **(✓the overall sample size calculation for DPHACTO has been reported before. For this specific study, the power/sample size calculation was based on available degree of freedom for the statistical analysis)** |
| Quantitative variables | 11 | Explain how quantitative variables were handled in the analyses. If applicable, describe which groupings were chosen and why **(✓page 9–10)** |
| Statistical methods | 12 | (*a*) Describe all statistical methods, including those used to control for confounding **(✓page 10–11)** |
|  |  | (*b*) Describe any methods used to examine subgroups and interactions (**N/A**) |
|  |  | (*c*) Explain how missing data were addressed (**N/A**) |
|  |  | *Cross-sectional study*—If applicable, describe analytical methods taking account of sampling strategy (**N/A**) |
|  |  | (*e*) Describe any sensitivity analyses **(✓page 11)** |
| Results | | |
| Participants | 13* | (a) Report numbers of individuals at each stage of study—eg numbers potentially eligible, examined for eligibility, confirmed eligible, included in the study, completing follow-up, and analysed **(✓page 12)** |
|  |  | (b) Give reasons for non-participation at each stage **(✓**Fig. 1**)** |
|  |  | (c) Consider use of a flow diagram **(✓**Fig. 1**)** |
| Descriptive data | 14* | (a) Give characteristics of study participants (eg demographic, clinical, social) and information on exposures and potential confounders **(✓**Table 1**)** |
|  |  | (b) Indicate number of participants with missing data for each variable of interest **(✓**Table 1**)** |
|  |  | (c) *Cohort study*—Summarise follow-up time (eg, average and total amount) **(N/A)** |
| Outcome data | 15* | *Cross-sectional study—*Report numbers of outcome events or summary measures **(✓**Table 1**)** |
| Main results | 16 | (*a*) Give unadjusted estimates and, if applicable, confounder-adjusted estimates and their precision (eg, 95% confidence interval). Make clear which confounders were adjusted for and why they were included **(✓pages 12–13,** Fig. 2**, page 9–10)** |
|  |  | (*b*) Report category boundaries when continuous variables were categorized |
|  |  | (*c*) If relevant, consider translating estimates of relative risk into absolute risk for a meaningful time period **(✓**Fig. 2**)** |
| Other analyses | 17 | Report other analyses done—eg analyses of subgroups and interactions, and sensitivity analyses **(✓page 14)** |
| Discussion | | |
| Key results | 18 | Summarise key results with reference to study objectives **(✓page 14)** |
| Limitations | 19 | Discuss limitations of the study, taking into account sources of potential bias or imprecision. Discuss both direction and magnitude of any potential bias **(✓page17)** |
| Interpretation | 20 | Give a cautious overall interpretation of results considering objectives, limitations, multiplicity of analyses, results from similar studies, and other relevant evidence **(✓page 14–17)** |
| Generalisability | 21 | Discuss the generalisability (external validity) of the study results **(✓page 14–17)** |
| Other information | | |
| Funding | 22 | Give the source of funding and the role of the funders for the present study and, if applicable, for the original study on which the present article is based **(✓page 19)** |
